# A Cohort Study on the Immunogenicity and Safety of the Inactivated SARS-CoV-2 Vaccine (BBIBP-CorV) in Patients With Breast Cancer; Does Trastuzumab Interfere With the Outcome?

**DOI:** 10.3389/fendo.2022.798975

**Published:** 2022-03-01

**Authors:** Maryam Joudi, Maryam Moradi Binabaj, Pejman Porouhan, Babak PeyroShabany, Mohsen Tabasi, Danial Fazilat-Panah, Mahtab Khajeh, Arezoo Mehrabian, Mansoureh Dehghani, James S. Welsh, Batol Keykhosravi, Azam Akbari Yazdi, Mona Ariamanesh, Ahmad Ghasemi, Gordon Ferns, Seyed Alireza Javadinia

**Affiliations:** ^1^Department of Pediatrics, School of Medicine, Zabol University of Medical Sciences, Zabol, Iran; ^2^Cellular and Molecular Research Center, Sabzevar University of Medical Sciences, Sabzevar, Iran; ^3^Department of Radiation Oncology, Vasei Hospital, Sabzevar University of Medical Sciences, Sabzevar, Iran; ^4^Department of Internal Medicine, Sabzevar University of Medical Sciences, Sabzevar, Iran; ^5^Center for Inflammation and Lung Research, Lewis Katz School of Medicine, Temple University, Philadelphia, PA, United States; ^6^Cancer Research Center, Babol University of Medical Sciences, Babol, Iran; ^7^Vasei Clinical Research Development Unit, Sabzevar University of Medical Sciences, Sabzevar, Iran; ^8^Student Research Committee, Iran University of Medical Sciences, Tehran, Iran; ^9^Department of Radiation Oncology, Neyshabur University of Medical Sciences, Neyshabur, Iran; ^10^Department of Radiation Oncology, Edward Hines Jr VA Hospital and Stritch School of Medicine, Loyola University Chicago, Chicago, IL, United States; ^11^Department of Pathology, Neyshabur University of Medical Sciences, Neyshabur, Iran; ^12^Department of Basic Sciences, Neyshabur University of Medical Sciences, Neyshabur, Iran; ^13^Department of Medical Education, Brighton and Sussex Medical School, Brighton, United Kingdom

**Keywords:** breast cancer, inactivated SARS-CoV-2 vaccine, seroconversion, SARS-CoV2, COVID-19 vaccine, cancer, malignancy, Iran

## Abstract

**Aim:**

To determine the efficacy and safety of inactivated SARS-CoV-2 vaccine (BBIBP-CorV) in patients with breast cancer.

**Methods:**

In this multi- institutional cohort study, a total of 160 breast cancer patients (mean age of 50.01 ± 11.5 years old) were assessed for the SARS-CoV-2 Anti-Spike IgG and SARS-CoV2 Anti RBD IgG by ELISA after two doses of 0.5 mL inactivated, COVID-19 vaccine (BBIBP-CorV). All patients were followed up for three months for clinical COVID-19 infection based on either PCR results or imaging findings. Common Terminology Criteria for Adverse Events were used to assess the side effects.

**Results:**

The presence of SARS-CoV-2 anti-spike IgG, SARS-CoV2 anti-RBD IgG, or either of these antibodies was 85.7%, 87.4%, and 93.3%. The prevalence of COVID-19 infection after vaccination was 0.7%, 0% and 0% for the first, second and third months of the follow-up period. The most common local and systemic side-effects were injection site pain and fever which were presented in 22.3% and 24.3% of patients, respectively.

**Discussion:**

The inactivated SARS-CoV-2 vaccine (BBIBP-CorV) is a tolerable and effective method to prevent COVID-19.

## Introduction

Breast cancer is the most commonly diagnosed cancer worldwide with a substantial public health burden as a result of approximately 2.3 million annual cases. in February 2021, the WHO announced that breast cancer has now overtaken lung cancer as the world’s most frequent cancer (1, 2). The COVID-19 pandemic has led to more than 219 million infections and 4.5 million deaths as of early October 2021 (3). The pandemic has also affected the cancer population, especially those with breast cancer. in multiple respects. Health services have been placed under great pressure, and in many countries with population breast mammography screening, efforts to diagnose and treat breast cancers have been restricted due to a lack of resources and priorities. There has been a delay in diagnosis and treatment, which may result in more patients with more advanced stages of disease, who will require more intensive treatment. This could result in an increase in morbidity and mortality. Figueroa et al, have documented this in an international evaluation of the impact of the pandemic on breast cancer early detection and screening on behalf of the Covid and Cancer Modelling Consortium (CCGMC) Working Group 2; demonstrating pauses in the national screening programs and stage shifting being reported in 9 countries (4).

In addition to the negative impact on patient treatment, the pandemic has also affected patient follow-up. Shortly after the outbreak, several large medical societies announced a consensus regarding how to prioritize different options for patients (5). Stratifying patients according to disease progression risks, viral exposure risks, and complications risks, as well as consideration of hospital resources, helped to minimize the disruption in patient care while preventing the spread of SARS-CoV-2. In order to delay the timing of surgery, neoadjuvant endocrine therapy for early-stage, hormone-positive malignancies was recommended (6–8). Wilke et al. described the impact of the COVID-19 pandemic on breast cancer management as “unprecedented”, and reported more personalized nonstandard approaches and forced pauses or delays in elective schedules (7). These negative impacts have been even more substantial in low- and middle-income countries (9–11).

The COVID-19 pandemic has also resulted in serious changes in cancer care within hospitals, with a decrease in oncology clinic admissions and an increase in viral pneumonia cases among cancer patients (12). In a study of 73 million patients in the USA, Wang et al. reported that patients with cancer had a greatly increased odds of COVID-19 infection (13). Additionally, cancer patients are at potential risk for more severe COVID19 infections, especially patients with breast cancer, because they are treated with treatment modalities and medicines that may alter the immune response (14, 15).

Although there is great interest in vaccinating cancer patient populations as a consequence of the reasons mentioned above and there are general recommendations regarding vaccinating in patients treated with different modalities and drug categories, there are no studies that confirm the safety and efficacy of vaccination in patients with different cancers and different treatments, as cancer patients have been excluded from COVID-19 vaccine trials so far (16, 17).

Here we report a cohort study on immunogenicity and safety of the inactivated SARS-CoV-2 vaccine (BBIBP-CorV) in patients with breast cancer, who were vaccinated as a part of a national plan for vaccination of patients with special diseases.

## Methods

In this multi-center cohort study, performed between March and June 2021, patients with cancer referred for vaccination to the cancer care network in the cities of Sabzevar, Neyshabur, and Babol were invited to participate. The protocol of the study was approved by the Ethics Committee of Sabzevar University of Medical Sciences (IR.MEDSAB.REC.1400.027) and a written informed consent form was obtained from the patients. Patients with acute conditions, including infection and immune-related complications, were excluded, and a written informed consent was obtained from all patients willing to participate in the study, or from their legal guardians. Previously, we reported the data on seroconversion rates and side effects of inactivated SARS-CoV-2 vaccine in a population of 364 cancer patients, and now we have analyzed their data to report the outcome of vaccination in breast cancer patients since they contributed the most (18).

The minimum sample size was estimated 144 people using the WHO sample size calculator for the evaluation of COVID-19 vaccine’s effectiveness based on results of Xia et al. (19) who reported 99% humoral responses against SARS-CoV-2 recipients received inactivated SARS-CoV-2 vaccine, desired precision width of 20%, and 30% of attack rate among unvaccinated individuals (19, 20).

Data about blood collection was discussed comprehensively in our previous studies (18, 21). Briefly, two doses of 0.5 mL Sinopharm β-propiolactone-inactivated COVID-19 vaccine (BBIBP-CorV), containing aluminum hydroxide-adjuvant were administered intramuscularly 28 days apart. Two months following the vaccination, blood samples were drawn to analyze the presence of SARS-CoV-2 anti-Spike protein (S) IgG and neutralizing antibodies. To evaluate the immunogenicity of the vaccine, the titers of SARS-CoV-2 Anti-Spike IgG, and SARS-CoV2 Anti RBD IgG were measured using two commercial ELISA kits [PISHTAZTEB DIAGNOSTICS, Tehran, Iran]. SARS-CoV-2 Anti-Spike IgG and SARS-CoV2 Anti RBD IgG were measured using the ELISA method according to the protocol described by the manufacturer (PISHTAZTEB DIAGNOSTICS, Tehran, Iran). Blood samples were collected from vaccinated patients and were centrifuged for 10 min at 1000 xg and stored for further analysis. Briefly, 100 μL of patients’ serum was added to 96- well plates (coated with CoV-2 Anti-Spike IgG or SARS-CoV2 Anti RBD IgG). After adding, the mixtures of the conjugate, plates were incubated at room temperatures. Following incubation, plates were washed and were emptied of wash buffer. Next, a chromogen substrate solution was added into the wells. After adding an equal volume of stopping solution to the wells, plates were read immediately at OD_450._ Both kits had good sensitivity, specificity, and accuracy (more than 99%). The cut-off value point for positive test in each of the two assays was 8 μg/ml (SARS-CoV-2 Anti-Spike IgG) and 2.5 μg/ml (SARS-CoV2-neutralizing antibody) as defined by the manufacturer. The sensitivity, specificity, and accuracy of both kits were 100% (95%CI 96.4-100), 99% (95%CI 94.9-99.9), and 99.5% (95%CI 97.4-99.9) respectively. Participants were followed for three months to evaluate the side-effects.

Data regarding local and systemic side-effects were collected weekly *via* telephone or in-person using a questionnaire based on the Common Terminology Criteria for Adverse Events (CTCAE) version V. Also, a hotline was established for the reporting of any serious acute side-effects. Moreover, all patients were followed-up for three months for clinical COVID-19 infection based on either PCR results or imaging findings. Common Terminology Criteria for Adverse Events were used to assess the side-effects.

Regarding the treatment protocols of the enrolled patients, in Iran, 1-year duration of trastuzumab given every three weeks (loading dose 8 mg/kg and then 6 mg/kg for a total of 17 cycles) is the current standard of care (22). Moreover, all of the patients on chemotherapy, received either dose-dense or every-three-week AC-T regiment (23). The hormone therapy was consisted of either tamoxifen or aromatase inhibitors.

Using SPSS 21, while reporting the descriptive analyses (frequency, percentage, mean, and standard deviation), data analysis was performed using Chi-Square and T-tests at the significance level of p<0.05.

## Results

A total of 160 breast cancer patients with a mean age of 50.01 ± 11.5 years (range 26 and 85 years) were included in the study, of whom 8 were male and 152 were female. The histologic type of all patients was invasive ductal carcinoma. Frequency of different stages was 2.5%, 28.1%, 60.0% and 9.4% for stage I, II, III, and IV, respectively.

Although only 15.6 percent of the patients (25 patients) had reported a history of previous COVID-19 infection, the prevalence of sero-positivity before vaccination, as assessed by serum SARSCoV2IgG levels, was 27.5% (n=44).

Two months after vaccination, 119 patients agreed to be tested for SARS-CoV-2 Anti-Spike IgG, and SARS-CoV2 Anti RBD IgG, however, the side-effects were assessed in all of 160 patients. The prevalence of positivity after vaccination was 85.7% (102 patients), 87.4% (104), and 93.3% (111) for SARS-CoV2-Spike Protein status, COVID19 Neutralizing Antibody status, and both, respectively. The prevalence of COVID-19 infection after vaccination was 0.7%, 0% and 0% for the first, second and third month of the follow-up period.

The frequency of different treatment modalities and approaches is shown in **Table 1**.

**Table 1:** Patients’ characteristics.

Variable	Frequency N (%)
**Stage**	
**I**	4 (2.5)
**II**	45 (28.1)
**III**	96 (60)
**IV**	15 (9.4)
**Histologic type**	
**Invasive ductal carcinoma**	160 (100)
**Molecular subtype**	
**Luminal A breast cancer**	44 (27.5)
**Luminal B breast cancer**	47 (29.3)
**Her 2 positive breast cancer**	34 (21.2)
**Triple negative breast cancer**	37 (22)
**Treatment modalities and approaches**	
**Chemotherapy**	35 (21.9)
**Radiotherapy**	28 (17.5)
**Follow up + Endocrine therapy**	44 (27.5)
**Follow up + Endocrine + Trastuzumab**	4 (2.5)
**Follow Up**	47 (29.4)
**Targeted therapy**	2 (1.3)

The efficacy of vaccination in terms of seropositivity, was further analyzed in subgroups regarding age, stage, and treatment (**Table 2**). Overall sero-positivity was not significantly different between the age and stage subgroups, although a significant difference was observed in patients <40 years of age in relation to the presence of COVID-19 neutralizing antibody positivity. An important finding was the significantly reduced efficacy of the vaccine in patients treated either with trastuzumab, or with chemotherapy. The rate of either SARS-CoV-2 Spike protein or Neutralizing Antibody positive result was only 75.0% in patients who were treated with trastuzumab, compared to 96.7% in patients of the follow-up group.

**Table 2:** Distribution of serologic responses following SARS-CoV-2 vaccination regarding patient characteristics, type of cancer, and treatment.

	SARS-CoV-2 Spike Protein Positive (%)	COVID-19 Neutralizing Antibody Positive (%)	Either SARS-CoV-2 Spike Protein or Neutralizing Antibody Positive (%)
**Age**			
**<40 years**	17 (73.9%)	16 (69.6%)	19 (82.6%)
**40-60 years**	71 (89.9%)	72 (91.1%)	76 (96.2%)
**>60 years**	14 (82.4%)	16 (94.1%)	16 (94.1%)
**P value**	0.143	**0.015**	0. 072
**Stage**			
**I**	3 (75.0%)	4 (100%)	4 (100.0%)
**II**	29 (87.9%)	29 (87.9%)	32 (97.0%)
**III**	64 (85.3%)	66 (88.0%)	69 (92.0%)
**IV**	6 (85.7%)	5 (71.4%)	6 (85.7%)
**P value**	0.917	0.526	0. 606
**Treatment 1**			
**Chemotherapy +/- RT**	18 (66.7%)	19 (70.4%)	21 (77.8%)
**Radiotherapy**	19 (95.0%)	19 (95.0%)	20 (100.0%)
**Follow up including Endocrine therapy, Trastuzumab**	65 (90.3%)	66 (91.7%)	70 (97.2%)
**P value**	**0.005**	**0.009**	**0.001**
**Treatment 2**			
**Follow up + Endocrine therapy**	35 (89.7%)	39 (100.0%)	39 (100.0%)
**Follow up + Endocrine + Trastuzumab**	3 (75.0%)	3 (75.0%)	3 (75.0%)
**Follow Up**	28 (93.3%)	25 (83.3%)	29 (96.7%)
**P value**	0.494	**0.020**	**0.014**

Bold text indicates a statistically significant difference with a p-value less than 0.05.

In terms of local side effects, the most frequent one observed here was mild injection site pain with 22.3% frequency. Low-grade fever (38-39°C) was the most frequent systemic side effect, which was documented in 24.3% of patients. More comprehensive data is provided in **Table 3**.

**Table 3:** Local and systemic side-effects following COVID-19 vaccination.

	Side-effect	Grading	Total (%)
**Local**	Pain	Mild	22.3
		Moderate	9.5
		Severe	2.7
	Swelling		3.4
	Itching		0.0
	Redness		6.8
**Systemic**	Fever	I (38-39°C)	24.3
		II (>39-40°C)	4.7
		III (>40°C ≤ 24 hours)	6.1
	Chills	I	9.5
		II	4.1
	Fatigue	I	17.0
		II	3.4
		III	0.7
	Anorexia	I	7.4
		II	3.4
	Nausea	I	7.2
		II	3.2
	Vomiting	I	2.0
		II	0.7
	Myalgia	I	17.6
		II	9.5
	Diarrhea	I	2.7
		II	0.0
		III	0.0
	Constipation	I	1.4
		II	1.4

## Discussion

Breast cancer has several subtypes based on estrogen receptor, progesterone receptor, and human epidermal growth factor receptor 2 (ER/PR/HER2) status (24).

COVID-19 is an infectious disease which has caused a global pandemic so that patients with cancer are at higher risk of infections owing to several factors such as chronic complications, overall weakened health status, use of systemic immunosuppressive medications, and anticancer therapy (25). There are several studies that show patients suffering from cancers are more susceptible to COVID-19 infection and its adverse consequences (26, 27). Moreover, it has been shown that patients with cancer are at a higher risk of morbidity and mortality following COVID-19 infection in comparison with healthy control groups (26). Exploring the associations between clinical manifestations of COVID-19 patients and serological responses, will provide us comprehensive insights toward understanding of the COVID-19 immunopathogenesis (28).

There are several decisive factors which play a crucial role in the management of COVID-19 in patients with breast cancer such as: the viral load of the virus, the pathogenesis of virus, comorbidities, age, and subsequent mortality (29). Also, there is a study showing that by increasing the COVID-19 pandemic period, the breast cancer mortality rate would increase 2 times in the next 2 years (30).

Different types of COVID-19 vaccines have been authorized by CDC and WHO and are widely used for the eradication of current pandemic. The BBIBP-CorV vaccine was created using inactivated particles of virus (killed vaccine), and was approved by the World Health Organization for emergency use (31). Evidence has been obtained from studies of vaccinations with BBIBP-CorV showing different side effects including fever, pain at the site of injection, and fatigue (32). Similar to previous studies, we found that mild injection site pain and low-grade fever were the most common side effects of COVID-19 vaccination (33).

Our results indicated that 85.7% and 87.4% of patients were positive for SARS-CoV-2 Anti-Spike IgG and SARS-CoV2 Anti RBD IgG, respectively. These results are in agreement with a study showed that 90% of patients were seropositive for SARS-CoV-2 anti-S IgG (34).

We also evaluated whether breast cancer treatment affected vaccination efficacy. In this regard, our results revealed that vaccination efficacy was significantly lower in patients treated either with trastuzumab or chemotherapy in comparison with those not receiving this treatment. This could be due to the immune suppressive effects of chemotherapy on immune function of cancer patients (35, 36). Previous findings suggested that chemotherapy can have profound and long-lasting negative effects on the bone marrow immune system for up to nine months after treatment and so leaving patients vulnerable to opportunity and secondary infections (37). So, a single booster dose of Covid-19 vaccine might be a beneficial approach to promote vaccine immunity performance after chemotherapy (38). Immunity response after co-administration of trastuzumab and chemotherapy has synergic effects meaning that in normal circumstances trastuzumab can improve humoral immune response but our study has a revers relationship with it (39).

In addition, our findings showed that trastuzumab can compromise the immune response after vaccination with BBIBP-CorV which could increase the risk of getting coronavirus or becoming very ill if patients get it. Besides, this risk can likely be higher if patients have a history of chemotherapy before treatment by trastuzumab.

Moreover, there was a decreased rate of COVID-19 infection after vaccination in breast cancer patients over the time. These results are in consistent with other studies showing the efficacy of COVID-19 vaccination in cancer patients in a time dependent manner (34, 40). In accordance with our results, there is a study showing a high rate of seroconversion in cancer patients, which indicates that those persons are able to provide sufficient antibody response to SARS-CoV-2 (41).

Our results also demonstrated that patient’s age (<40 years) was associated with lower seropositivity (Neutralizing Antibody Positivity). On the other hand, Lacono and colleagues reported that, none of patients with cancer who were aged 80 years or older became infected with COVID19 regardless of IgG response (42). In addition, there are studies suggesting that in the general population, the number of detectable antibodies in common human coronaviruses is increased with increasing age (43, 44). However, in another study it was reported that decreasing in detectable antibody was not necessarily associated with low immunity, and the naturalization capacity is equal to those with higher quantity of antibody (45).

The results of current study shows that only 15.6% of participant reported a positive test of COVID-19, while the prevalence of seropositivity before vaccination was 27.5%. This could be due to the asymptomatic SARS-CoV-2 infections in patients showing no COVID-19 related symptoms until clinical diagnosis (46, 47). Moreover, patients who were referred for specialist care to hospitals and clinics during the COVID-19 pandemic are lower in comparison with pre-pandemic period, because of the risk of possible exposure (48).

Our results showed that there was a good vaccine efficacy in patients with breast cancer. However, it is important to note that there is a need for further research and larger studies over longer periods in order to generate more convincing evidence on vaccine efficacy in vulnerable populations, including patients with cancer, to fully assess the safety, potential benefits and risks of COVID-19 vaccination.

Our study may have been affected by several biases. First, the low number of patients who undergo serological testing after being vaccinated and. Second, a longer follow-up is needed to determine the duration of the serologic response. Third, the present study only includes patients from only one hospital. Fourth, there was no control cohort of cancer patients who had not been vaccinated against COVID-19. Moreover, the number of patients receiving trastuzumab alone was small which makes it difficult to interpret results.

Taken together, obtained results confirm that approved vaccines can help patients with cancer to protect themselves from COVID-19 infection. These findings highlight the importance of early vaccination in diseases with high risk including cancer, especially patients who are currently receiving treatment for cancer that may arise from various complications contributing to COVID-19. As vaccination is increasing rapidly in many countries, and more data becomes available in context from reliable sources, more solutions for vaccination challenges will be available.

## Conclusion

The inactivated SARS-CoV-2 vaccine (BBIBP-CorV) is well tolerated and efficacious intervention to prevent COVID-19.

## Data Availability Statement

All data generated and analyzed during this study can be accessed through direct communication with the corresponding author and the agreement of all research team members.

## Ethics Statement

The studies involving human participants were reviewed and approved by Sabzevar University of Medical Sciences (IR.MEDSAB.REC.1400.027). The patients/participants provided their written informed consent to participate in this study.

## Author Contributions

Study concept and design: SAJ, MD and MA; acquisition of data: MA, PP, BP, MT, DF, BF, MKh; analysis and interpretation of data: AAY, SAJ and MJ; drafting of the manuscript: MMB, AM and AGh; critical revision of the manuscript for important intellectual content: MT, GF, JSW, SAJ; statistical analysis: SAJ and MJ. All authors contributed to the article and approved the submitted version.

## Conflict of Interest

The authors declare that the research was conducted in the absence of any commercial or financial relationships that could be construed as a potential conflict of interest.

## Publisher’s Note

All claims expressed in this article are solely those of the authors and do not necessarily represent those of their affiliated organizations, or those of the publisher, the editors and the reviewers. Any product that may be evaluated in this article, or claim that may be made by its manufacturer, is not guaranteed or endorsed by the publisher.
